# Influence of language on perception and concept formation in a brain-constrained deep neural network model

**DOI:** 10.1098/rstb.2021.0373

**Published:** 2023-02-13

**Authors:** Malte R. Henningsen-Schomers, Max Garagnani, Friedemann Pulvermüller

**Affiliations:** ^1^ Department of Philosophy and Humanities, Brain Language Laboratory, Freie Universität Berlin, Habelschwerdter Allee 45, 14195 Berlin, Germany; ^2^ Department of Computing, Goldsmiths, University of London, London, SE14 6NW, UK; ^3^ Berlin School of Mind and Brain, 10099 Berlin, Germany; ^4^ Einstein Center for Neurosciences, 10117 Berlin, Germany; ^5^ Cluster of Excellence ‘Matters of Activity. Image Space Material’, Humboldt-Universität zu Berlin, 10099 Berlin, Germany

**Keywords:** concepts, linguistic relativity, cognition, Hebbian learning, neurocomputational modelling, deep neural networks‌

## Abstract

A neurobiologically constrained model of semantic learning in the human brain was used to simulate the acquisition of concrete and abstract concepts, either with or without verbal labels. Concept acquisition and semantic learning were simulated using Hebbian learning mechanisms. We measured the network's category learning performance, defined as the extent to which it successfully (i) grouped partly overlapping perceptual instances into a single (abstract or concrete) conceptual representation, while (ii) still distinguishing representations for distinct concepts. Co-presence of linguistic labels with perceptual instances of a given concept generally improved the network's learning of categories, with a significantly larger beneficial effect for abstract than concrete concepts. These results offer a neurobiological explanation for causal effects of language structure on concept formation and on perceptuo-motor processing of instances of these concepts: supplying a verbal label during concept acquisition improves the cortical mechanisms by which experiences with objects and actions along with the learning of words lead to the formation of neuronal ensembles for specific concepts and meanings. Furthermore, the present results make a novel prediction, namely, that such ‘Whorfian’ effects should be modulated by the concreteness/abstractness of the semantic categories being acquired, with language labels supporting the learning of abstract concepts more than that of concrete ones.

This article is part of the theme issue ‘Concepts in interaction: social engagement and inner experiences’.

## 1. Introduction

Are words merely handy tools which, by providing convenient ‘labels’ for concepts, allow for more efficient communication? Or do words as linguistic symbols have a more profound wide-ranging influence on conceptual development and semantic processing?^1^ This question can be viewed as part of the more fundamental question as to whether cognition can be viewed as modular [1], with different independent cognitive modules that are informationally encapsulated from each other, and processing of concepts being independent from that of their corresponding symbols, or whether different cognitive faculties such as language, perception and conceptual processing rely on at least partially shared neural substrates and therefore might also functionally interact and depend on each other [2–4].

In recent years, two aspects have received particular attention; on the one hand, the Sapir-Whorf hypothesis [5], also known as the linguistic relativity hypothesis (for reviews, see [6–8]), predicts that a speaker's language, specifically the way in which this language ‘carves up’ and categorizes percepts, influences how those percepts are processed to start with. This has most extensively been studied in the domain of colour where an impressive body of evidence now exists suggesting that differing colour vocabularies in different languages are linked with different performance in colour perception, categorization and memory, both at the behavioural and neural levels [9,10]. Recent work has extended these findings to tactile stimuli [11,12], odours [13] and motion and event perception [14–18]. Crucially, experiments on perceptual learning could directly confirm a causal effect of the presence of consistent ‘verbal labels’ on perception in the same individuals [11,12,19].

The linguistic relativity debate can be viewed as part of a more general discussion concerning the question of whether perception and higher cognition, including conceptual categorization, interact, in particular, whether there are ‘top-down’ influences of cognition on perception [20,21]. If so, this would question a strictly modular distinction between cognition and perception with functionally separate processing components [22]. Effects of linguistic relativity, for example, the relatively better perceptual discrimination of visual or tactile stimuli that have been learnt in context of consistent verbal labels, can tentatively be explained by causal and functional interaction between perceptual and linguistic representations. In this context, Lupyan proposed the term ‘label feedback hypothesis' [23–25] and argues that labels ‘play an active role in perception and categorization by selectively activating perceptual features that are diagnostic of the category being labelled’ [24, p. 4]. However, in order to explain the emergence of such a link between linguistic and perceptual properties, a biologically plausible cognitive-computational model is necessary. Lupyan presents a connectionist model implementing a 30-unit 'perceptual' layer and a 2-unit 'label' layer, with a 60-unit hidden layer between them along with learning driven by the technically efficient backpropagation rule [26]. He shows that, within this model, labels help sharpen categorical perception, i.e. exemplars belonging to the same category (with the same label) are perceived as more similar to each other and those belonging to different categories as more dissimilar to each other [26]. However, we see several shortcomings in this work: (i) the model used was a connectionist model with fully distributed representations and no connections within ‘layers’, features that contrast with the frequently assumed sparseness of cognitive representations and strong within-area connections in the cortex [27]; (ii) the acquisition of only two concepts was simulated, and for each concept, only a single prototype was used during learning, which is cognitively not very plausible, as humans can experience a wide range of different object instances when learning the meaning of a linguistic object label; (iii) Lupyan's model is applied to concrete object related concepts and their ‘labels’, whereas the emergence and language-relatedness of abstract concepts, which do not simply ‘label’ objects, remain unaddressed; and (iv) Lupyan's associative model of three layers is far removed from the structure and connectivity of the cortical areas contributing to the processing of symbols, perceptions, actions and concepts. A few other earlier neural network studies had also investigated concept formation (e.g. [28,29]), but the same caveats which we mentioned in relation to Lupyan's model [26] apply and any conclusions suggested by them might still need modification in view of more realistic simulation studies [27]. We therefore think it is worthwhile to take a closer look at the putatively underlying mechanisms and ask how semantic and conceptual representations develop in a neuroanatomically and neurophysiologically more realistic architecture receiving information about the co-occurrence of linguistic expressions and the perceptions and actions which these ‘labels’ relate to, and applying a learning algorithm consistent with synaptic dynamics observed in neurobiological research.

In the developmental psychology literature, the role of concrete verbal labels in conceptual acquisition in infants has long been a subject of interest as well, with a plethora of evidence suggesting that labels play an important role in normal concept acquisition (see [30] for review). For example, Waxman & Markow [31] argue that labels are ‘invitations to form categories' and help to highlight similarities between objects belonging to the same semantic category (and hence being referred to with the same word). This does not imply that it is always strictly *necessary* to associate a label with a concept; for example, Pinker [32] argues that even non-German speakers who are not familiar with the German word ‘Schadenfreude’ seem to have a concept for this socially established emotion nonetheless, even before finding out that a single word for this concept exists (in a different language). A related example given by Pinker—with only anecdotal evidence—is that many people would agree that they have a concept of dust that has accumulated into a dust ball under a bed despite not having an apt label for this. In addition to such anecdotal evidence for concepts that supposedly exist before and independent of any language, a recent study with patients suffering from organic language impairments, aphasias, claimed that despite their language impairments they performed normally on a categorization task [33], thus suggesting that language is not necessary for categorization. However, other studies [34–36] had previously reported that aphasic patients were indeed impaired in categorization tasks, even if these tasks themselves were purely non-verbal. Caution is required in interpreting these data, however: although lesion loci causing aphasic deficits (posterior inferior frontal and superior temporal cortex) may dissociate from those sites where the most pronounced problems with conceptual tasks have been reported after lesion (anterior inferior temporal cortex) (e.g. [37]), these lie in close vicinity; also, additional cortical areas are important for concept processing [4,38]. Furthermore, and most importantly, the presence or absence of a conceptual distinction in aphasic patients who do not speak overtly is no proof of entirely absent language ability in these individuals, and hence cannot be interpreted as evidence for language-dependent or -independent conceptual processing.

In summary, the evidence in favour of language-independent concept formation appears to be either on weak empirical grounds or anecdotal. On the other hand, the strong position that labels are strictly necessary for category learning is not fully supported either. However, it seems that, at the very least, labels improve or guide category learning in important ways. For example, a number of studies indicate that labels guide infants' attention to perceptual features common to many objects that fall under a given category, therefore promoting concept formation [39,40]. As Dove puts it, ‘the act of labelling […] may help learners become attuned to perceptual commonalities and overcome the inherent complexity and noisiness of perceptual inputs’ [41, p. 4]. However, whereas theoretical proposals postulate, and some experimental data support, ‘guidance’, ‘shaping’ or ‘warping’ of attention, category building or perception, the mechanisms underlying such linguistic conceptual effects remain poorly specified or restricted to simple cases, in particular to concrete words. How—and based on which mechanisms—could such guidance toward common object features come about? Also, how could any concrete mechanism account for the formation of abstract concepts?

**Figure 1.**
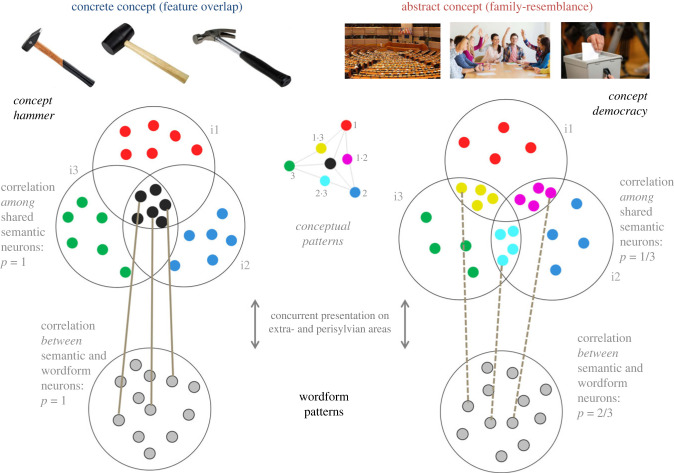
Adapted from [46]. Schematic illustration of a structural difference between concrete (left) and abstract (right) concepts (semantic feature overlap versus family resemblance). We model the semantic features of any given concept as shared neuronal elements of three ‘grounding patterns’, with 12 neurons per grounding pattern and modality (sensory/visual and motor, i.e. 24 per grounding pattern across both modalities). Only one modality is shown for clarity; procedures were identical for grounding patterns used as input to the visual and motor ‘cortices’ of the model (see figure 2). Top left panel: concrete concepts were modelled as containing 12 neurons per grounding pattern in total, six shared between all three (therefore representing semantic features) and six unique to each instance (representing instance-specific perceptual or action-related features). In the example of HAMMER, the six shared and therefore ‘semantic’ neurons represent general visual features such as shape features including long handle, head attached at a 90 degree angle along with general action-related ones, including typical motor trajectories characterizing the beating with a hammer. The six instance-specific sensory and motor neurons represent unique features of each hammer exemplar including idiosyncratic properties (e.g. differing sizes, materials, shapes of the head, presence or absence of a wedge), along with specificities of the way each hammer requires sensorimotor adjustment to these individual properties when being used. Top right panel: abstract concepts were modelled by an implementation of family resemblance, whereby each grounding pattern of an instance is represented by 12 neurons, four shared between two instances and four unique to only one instance. In the example of DEMOCRACY, pairwise shared neurons might represent hand actions involved in casting a vote (shared between i2/i3) or the visual image of several people coming together (shared between i1/i2). Unique features might represent differences in the hand movements for raising one's hand versus throwing a ballot in a ballot box (i2 versus i3) or differences in the size and layout between an official parliament room and a smaller room where people cast votes in an informal setting. Bottom panel: all wordform patterns (supplied as input to perisylvian areas during training in the label conditions) consisted of 12 neurons per pattern that were always identical for all three instances of a concept. Brown lines: illustration of the correlation structure (i) among shared neurons in conceptual grounding patterns and (ii) between these shared neurons and the neurons of wordform patterns. For illustration purposes, the correlations are illustrated with examples (brown solid and dashed lines). Whereas for concrete concepts (left), the average correlation is *p* = 1 both among shared-conceptual and between shared-conceptual and wordform neurons, for abstract concepts there is a higher correlation between shared neurons and word form neurons (*p* = 2/3, as the wordform always co-occurs with two thirds of the set of neurons formed by the union of all pairwise-shared neuronal sets) than among shared neurons (*p* = 1/3, as any two concepts share only a third of such a union set). Hence, this difference in correlations—which is present for abstract concepts only—might exert a ‘pull’ on the emerging cell assemblies during training, such that the word form neurons, because of their relatively higher correlation, end up playing a more important role in the entire cell assembly's structure (and, hence, dynamics). See the Introduction for a detailed discussion. Photographs were obtained from the world wide web and were published under a CC0 license (https://creativecommons.org/share-your-work/public-domain/cc0/).

When it comes to explaining crucial differences between concrete and abstract concepts, a range of authors make purely quantitative claims, for example, that the number of total semantic features [42] or the proportion of shared semantic features is relatively larger for concrete concepts [36,43,44]. However, as discussed elsewhere [45,46], we believe this approach is too superficial and fails to capture the nature of abstract concepts. Consider concepts such as DEMOCRACY or GAME, where certain semantic features only apply to subgroups, but not to all members of the concept. Hence, although we agree that in general, the purely quantitative distinction (fewer features shared by all members of abstract than concrete concepts) made by many authors is correct, this description misses the important point that the degree of ‘sharedness’ of semantic features differs. Therefore, we here adopt the view that abstract concepts do not normally have many semantic features shared across all their category members. Instead, these show a pattern of family resemblance [45,47–49], where features are not shared across all category members but just across a subset (see also figure 1 for illustration). Property generation tasks [50–52] show that abstract concepts are much more dependent on context than concrete ones and that a much more variable set of features characterizes the situations (objects, actions etc) associated with the concepts; we interpret this as indirect evidence that modelling abstract concepts as being characterized by a family resemblance structure is appropriate, although we acknowledge that a systematic empirical investigation on this question is still needed. This approach, which we implemented and discuss in detail in a recent simulation study [46], provides a straightforward biological explanation for why concrete and abstract concepts are processed differently in the mind and brain: the frequently occurring shared perceptual (or action-related^2^) features of the instances of concrete concepts are activated together whenever prototypical category members are being processed; assuming that each perceptual or action-related feature has a neuronal basis, this leads to strong binding between the neurons involved, thereby leading to the formation of neural category representations. By contrast, the variable and non-overlapping perceptual features activated in processing the more heterogeneous instantiations of abstract concepts activate not-fully overlapping neural representations so that there is less opportunity to bind together the feature neurons contributing to an abstract concept according to biological learning mechanisms. Previous simulations [46] showed that the biological correlates of concrete categories emerged spontaneously during the processing of similar objects or actions that fall under a concrete concept. However, for abstract concepts, the formation of functionally coherent category representations was more fragile. We here ask whether the addition of labels, a feature of human conceptual development, significantly influences the formation of the neurobiological correlates of concrete and abstract concepts and whether there are differences in such influences for the two main conceptual types. We expect that the consistent perception of a wordform during concept formation will improve the building of a category representation for concrete concepts but will be even more beneficial for building abstract categories. We here focus on direct conceptual grounding only but do acknowledge that after a grounding kernel of concrete and abstract concepts has been acquired by direct grounding (or ‘word-world-correlations mapping’), additional new concrete and abstract concepts can be learned through indirect grounding via distributional associations (word-word-correlations mapping) [47,53–55].

From a theoretical neurobiological perspective, the formation of concepts and categories can be founded in neuronal learning. Biologically realistic neuronal learning translates the strength of correlated neuronal activation into the strength of the connections of the partaking (and previously already connected) nerve cells. We take the ‘neurons’ schematically shown in figure 1 to represent information about the relevant word forms and semantic features of the concepts. Briefly, because the shared features of all the hammers (e.g. all have a handle, can be grasped and have a heavy head) are active whenever any hammer object is processed together with the related word ‘hammer’, there will be strong links between shared conceptual/semantic feature neurons and the word form representation. This link will be weaker for abstract concepts because each of the partly shared semantic feature neurons is active only for a subset of the available instances (raising one's hand or expressing one's opinion is relevant for decision making in only some instances of democracies, not for example in parliamentary democracies, where a cross has to be made every few years and most decisions are taken by delegates). Therefore, the abstract word form representation co-activated while processing instances of democracies will lead to relatively weaker binding between word form and the partially shared semantic-conceptual features. Still, the association of a ‘label’ with all the partly shared semantic feature neurons will interlink these partially shared feature neurons more strongly with each other, thus potentially facilitating the formation of a coherent concept. Therefore, from a theory-driven neurobiological perspective and under certain assumptions, the addition of word forms to both types of concepts can produce a significant advantage in building categories; this effect may be most significant for abstract ones, as their previously fragile neuronal representations become interlinked by the word form neurons. In this case, the word forms act as the computational ‘glue’ that holds together the concept. Nonetheless, it remains unclear whether—and if so, to what extent—the result of this ‘toy’ example would transfer to a large-scale, brain-like model, in which the number, connectivity among, and complex nonlinear physiological features of the component elements more closely approximate those of the real cortex.

**Figure 2.**
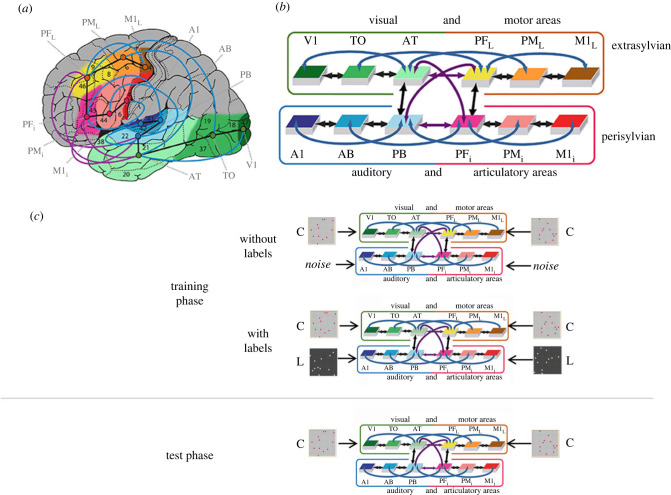
Panels (*a*) and (*b*) adapted from [46]. (*a*) Structure and connectivity of the neural network model. Twelve brain areas were modelled in total, including areas in frontal, temporal and occipital cortex. Perisylvian areas comprise an inferior-frontal articulatory (red colours) and a superior temporal auditory system (blue colours), and extrasylvian areas comprise a lateral dorsal hand-motor system (yellow/brown) and a visual ‘what’ stream of object processing (green). Numbers refer to Brodmann Areas (BAs) and the arrows represent long distance cortico-cortical connections as documented by neuroanatomical studies (see table 2 in [56] for neuroanatomical evidence). (*b*) Schematic depiction of the brain areas modelled (using the same colouring for different brain areas as in panel (*a*), along with their connectivity structure. The different colours of arrows (black, blue, purple) stand for ‘next-neighbour' connections linking cortically adjacent areas within each system (black arrows) and ‘jumping links’ between non-adjacent cortical areas within each system (blue links) as well as ‘long distance links’ between pairs of multimodal areas *PB, *PF_i_, *AT and *PF_L_ (purple links). (*c*) Illustration of the training phase (top) and testing phase (bottom). In the training phase, there were two conditions, either without labels or with labels. In the no label condition, noise instead of wordform patterns were presented to perisylvian primary areas as input. Input of conceptual patterns (marked with a ‘C’ for illustration) was always given to the primary extrasylvian areas (top row) and input of wordform patterns (marked with ‘L’ if present) was given to the primary perisylvian areas (bottom row). Note that the differential colouring and marking with ‘C’/’L’ of input patterns is for illustration purposes only and is not information that was explicitly given to the model.

For investigating the role of word forms and language in the formation of concrete and abstract concepts, we chose to build a neurobiologically constrained neural network model of several areas of the human cortex that are known to be involved in language and conceptual processing. The model used for simulating the brain mechanisms of conceptual and semantic learning included model analogues of those brain regions that are involved in processing perception and action-related information which may give rise to category formation. These are visual areas, through which sensory information about the surrounding world comes in, as well as motor systems, which are essential for action processing (other sensory modality systems were omitted to keep the model manageable). For each of the systems—visual and motor—three cortical areas were modelled, one primary (M1_L_ and V1), one secondary (PM_L_, TO) and one ‘higher’ area (PF_L_, AT) with strong links to other systems. We asked whether the perception of similar objects and the execution of similar actions would give rise to the formation of neuronal representations for object/action categories. In addition, to implement the learning and processing of symbols, i.e. spoken words, the articulatory motor cortex (M1_i_) and the auditory cortex (A1) was modelled, again with secondary and higher areas for each system (PM_i_, PF_i_ and AB, PB). This allowed us to first study the formation of concepts based on sensory input (object perception, stimulation to V1) and action execution (to M1_L_, see also [46]) and then, second, to investigate the influence of word form learning (triggered by activity in A1 and M1_i_) on concepts when these merge into symbolic semantic circuits. Therefore, our choice of areas was oriented towards the to-be-addressed problems but also aimed at a maximum of neurobiological realism, insofar as seven constraints recommended for biologically realistic neural modelling [27] were implemented (in contrast to standard deep neural networks which do not tailor network structure to the research question and are not explicitly oriented towards modelling specific brain parts and areas and their connectivity). Note once again that the only areas which receive patterns as direct input during training are the primary sensory/motor areas (figure 2*c*).

Using the same approach to simulating semantic category learning as in a recent simulation study [46], we created ‘grounding patterns’ of neuronal activity each thought to represent an object and/or a related action. A concept was thought to be grounded in three related individual objects (and object representations), also called ‘instances’ of the concept. Each grounding set thus consisted of three grounding patterns, whereby triplets of patterns showed different similarity structures for concrete and abstract concepts, exhibiting either full sharing of neuronal elements or family resemblance. Our strategy here was to simulate the perceptuo-motor processing of conceptual instances. During a learning phase, these conceptual instances were either associated, through Hebbian learning mechanisms, with linguistic labels, or not, allowing us to investigate possible causal ‘Whorfian’ effects of language on conceptual and perceptual processing in the model. Based on the outlined neurobiological language model, we hypothesize that (i) concrete semantic category formation is slightly improved by the addition of wordforms denoting the category, whereas (ii) abstract semantic category formation is deficient without any interplay with verbal language, but substantially improved by the addition of wordforms for the semantic categories.

## 2. Methods

Building on earlier modelling work [46,56–58], we used a neuroanatomically grounded, neurophysiologically plausible computational model with spiking neurons and 12 model areas representing visual and motor as well as auditory and articulatory areas in frontal, temporal and occipital along with adjacent multimodal hub areas cortices that are known to be important for processing words and their meaning.

### (a) Model architecture^3^

We adopted a model architecture constrained by neurobiological information and previously applied to explore neural mechanisms of semantic learning [46,56,57,59,60]. The following brain constraints were applied to the model (for a recent review of this brain-constrained modelling approach, see [27]):

(i) neurophysiological dynamics of spiking pyramidal cells including temporal integration (summation) of inputs, threshold-based probabilistic spiking and adaptation [61,62] were implemented (following [56,58]);
(ii) synaptic weights were modified by way of unsupervised Hebbian-type learning, including both long-term potentiation (LTP) and long-term depression (LTD) [63] (following [58]);
(iii) global and local activity regulation [64,65] and control were realized by area-specific and local inhibition (following [66]);
(iv) 12 areas commonly distinguished in inferior and dorsolateral frontal, superior temporal and ventral temporal and occipital cortex were modelled (following [60]);
(v) within-area connectivity included local excitatory and inhibitory connections (see also (iii)) excitatory connections were sparse, random and initially weak exhibiting a neighbourhood bias toward close-by links [67,68] (following [69]);
(vi) between-area connectivity was implemented in accordance with neuroanatomical studies (following [59,70]; for a review of the evidence on all implemented connections, see table 2 in [56]); and
(vii) inherent baseline noise (white noise) was constantly present in all neurons of all areas during learning and while recording the network response to learnt patterns. In addition, perisylvian areas not receiving a specific pattern as input during learning received further uncorrelated white noise activation to simulate variable inputs (following [57,60]).

For further details about the implementation, including the equations implemented in the simulation software used, the reader is referred to the Appendix in [46]. The parameters used in the simulation are described in table 1.

**Table 1.** Parameter values used for the simulations. (Equation numbers refer to the equations in the appendix of [46], where further details about the mathematical implementation of the model are described.)

eqn (1)	time constant (excitatory cells)	*τ* = 2.5 (time steps)
	time constant (inhibitory cells)	*τ* = 5 (time steps)
	total input rescaling factor:	*k*_1_ = 0.01
	noise amplitude:	*k*_2_ = 7·√(24/Δt) (Δt = 0.5 ms)
	global inhibition strength:	*k*_G_ = 0.80
eqn (3)	spiking threshold	thresh = 0.18
	adaptation strength	*α* = 8.0
eqn (4)	adaption time constant	*τ*_ADAPT_ = 10 (time steps)
eqn (5)	rate-estimate time constant	*τ*_Favg_ = 30 (time steps, training)
		*τ*_Favg_ = 5 (time steps, testing)
eqn (6)	global inhibition time constant	*τ*_GLOB_ = 12 (time steps)
eqn (7)	postsynaptic potential thresholds for	ϑ_+_ = 0.15 (LTP)
		ϑ_−_ = 0.14 (LTD)
	presynaptic output activity required for any synaptic change:	ϑ_pre_ = 0.05
	learning rate:	Δw = 0.0008

### (b) Simulated brain areas and their connectivity structure^4^

The spiking network model mimicked 12 different cortical areas with area-intrinsic connections and mutual connections between them (figure 2*a*,*b*). Note that we refer to model brain areas using an asterisk (e.g. *V1). Six areas were modelled for the left-perisylvian language cortex including the primary auditory cortex (*A1), auditory belt (*AB) and modality-general parabelt areas (*PB) constituting the auditory system, and the inferior part of primary motor cortex (*M1_i_), inferior premotor (*PM_i_) and multimodal prefrontal motor cortex (*PF_i_) representing the articulatory system (i.e. inferior face-motor areas). Additionally, six extrasylvian areas were modelled including the primary visual cortex (*V1), temporo-occipital (*TO) and anterior-temporal areas (*AT) for the ventral visual system and the dorsolateral fronto-central motor (*M1_L_), premotor (*PM_L_), and prefrontal cortices (*PF_L_) for the dorsolateral action system.

The network's between-area connectivity structure reflects existing anatomical pathways between corresponding cortical areas of the cortex revealed by neuroanatomical studies using diffusion tensor and diffusion-weighted imaging in humans and non-human primates that are discussed in detail in a previous study [56,58] and summarized in table 2 of Tomasello *et al*. [56]. In summary, these anatomical pathways were modelled between adjacent cortical areas within each of the four ‘streams’ (see black arrows in figure 2*a*,*b*) and between all pairs of multimodal areas (*PB, *PF_i_, *AT and *PF_L_) through the long distance cortico-cortical connections (purple arrows). Additionally, as a previous neurocomputational study [70] demonstrated the importance of non-adjacent ‘jumping’ links for verbal short-term memory, such second-next-neighbour-links (skipping one intermediate area) were also included within the superior and inferior temporal and the superior and inferior frontal processing streams (blue arrows).

### (c) Conceptual grounding patterns^5^

In the present simulation, conceptual grounding patterns were used as input to extrasylvian brain areas during training and wordform grounding patterns were used as input to perisylvian brain areas. The conceptual grounding patterns were identical to the ones used in a recent publication [46]. In order to model effects related to semantic category learning, we created sets of grounding patterns each thought to represent one object and/or action. For each semantic concept, we created three grounding patterns, whereby triplets of patterns showed different similarity structures for concrete and abstract concepts, exhibiting either full sharing of neuronal elements or family resemblance (figure 1). There were 10 concepts per semantic category (abstract/concrete), and thus 30 instances of grounding patterns overall for each semantic category type. For example, the concept of HAMMER would be represented by three grounding patterns of hammers differing in size, shape, material etc. and hence each one having somewhat differing perceptuo-motor experiences. An abstract concept like DEMOCRACY would similarly be represented by three grounding patterns representing the image and/or experience of a parliament building, a polling booth and a situation where people vote by raising their hands (for illustration, figure 1).

Each (simulated) grounding pattern consisted of 12 ‘active’ cells in *V1 and 12 *M1_L_ each (i.e. 12 ‘active’ out of the possible 625 neurons per area). Patterns were designed in such a way that each neuron occurred in only one concept, i.e. overlap always only occurred within the three instances of one concept, but never across the 10 different concept patterns used. Furthermore, different models were used for concrete and abstract simulations (i.e. each individual model was trained with either concrete or abstract grounding patterns only). This allowed us to use identical wordform patterns for both concrete and abstract concepts, ruling out any possible differences between the set of wordform patterns used as confound. A further reason for using different models for concrete and abstract concept simulations was to avoid possible interferences between conceptual types. We here follow the idea that concrete concepts have *feature overlap neurons* which all instances of a grounding pattern representing a concept share, while for abstract concepts, there are no neurons common to all three instances, but only neurons shared by 2 out of 3 instances (i.e. pairwise shared neurons), resulting in a *family resemblance structure*. In addition, both concrete and abstract concepts also had *unique neurons* occurring in only one grounding pattern. For a detailed discussion of this approach of modelling the conceptual grounding patterns for concrete and abstract concepts, see Henningsen-Schomers & Pulvermüller [46].

### (d) Labelling and wordform patterns

Apart from the semantic factor (concrete versus abstract conceptual patterns), which has been studied in more detail elsewhere [46], we introduced a further factor in the present simulations, that of wordform labelling. That is, each model was not only run in a version where it only received conceptual grounding patterns as input to extrasylvian areas (‘no label’ condition), but we also ran each model in a condition where, simultaneously with the inputs presented to extrasylvian areas, ‘wordform’ patterns were active on the perisylvian model areas (‘label’ condition). These wordform patterns can be thought of representing a linguistic label, hence causing the model to associate a wordform (or label) with the conceptual information. Such association takes place by virtue of the presence of Hebbian learning. Just like the conceptual grounding patterns, wordform patterns also consisted of 12 ‘active’ cells, i.e. 12 each to be used as input to *A1 and *M1_i_. The wordform patterns for a given concept (consisting of three varying, but related conceptual patterns) were always entirely identical.

### (e) Training procedures

Our simulations had a 2 × 2 factorial design with factors semantic-type (concrete/abstract) and labelling (label/no label), resulting in four conditions in total. We ran a total of 12 instantiations of the model for each condition (the model correlate of running 12 human participants in an experiment), each with identical training patterns and procedures, hence 48 models in total. To implement the equivalent of some random variation as would be present across individual human participants, we randomized for each model all synaptic links (and corresponding weights) between cells in connected areas (and within areas) before training (model initialization). The same set of initial randomized synaptic links and weights was then used to train a model with concrete patterns and with abstract patterns but in separate model instances. Separate instantiations were used for the learning of concrete and abstract concepts to avoid interference between the two types of conceptual representations.

In the ‘no label’ conditions, each training trial consisted of randomly choosing one of the 30 sensorimotor patterns (consisting of 12 ‘active’ neurons per area, described in detail above) and presenting it as input to extrasylvian primary areas (*V1 and *M1_L_) continuously for 16 time steps. No correlated input was given to the ‘phonological’ part of the model (perisylvian primary areas *A1 and *M1_i_) in this case. Instead, uncorrelated white noise stimulation was applied to these at all times. However, in the ‘label’ condition, additional input of the wordform patterns was given to the perisylvian primary areas (*A1 and *M1_i_), also continuously for 16 time steps.

To avoid potential contamination between successively presented stimulus patterns, an interstimulus interval (ISI) followed each pattern presentation. This ISI lasted until global inhibition in areas *A1 and *PB had returned below a specific threshold so that network activity had returned to a baseline value to prevent one trial from affecting the next one. During these ISIs network activity was driven entirely by uniform white noise, simulating the spontaneous baseline neuronal firing observed in real neurons. Instead of stimulus patterns, additional (environmental) white noise was also presented as input to all primary model areas (*V1, *M1_L_, *A1, *M1_i_) during ISIs. Training continued until 4000 repetitions of each instance of a pattern had occurred, i.e. 12 000 repetitions per concept.

### (f) Testing procedures

After learning, a testing phase was implemented to examine the result of learning. This testing phase was always identical for all four conditions of the 2 × 2 design. Hence, any differences observed between conditions are a result of the different patterns presented during the training phase, and cannot have arisen in the testing phase. Note that during the testing (or so-called ‘read-out’) phase no learning in the network was allowed. Because the aim of our project was to investigate a ‘Whorfian’ influence of linguistic labels on perceptual processing in the absence of linguistic input, in testing we only stimulated the extrasylvian model areas with the previously learnt conceptual grounding patterns and recorded the model activity (see details below). Hence, during the testing phase—which all analysed results are based on—the perisylvian part of the model did not receive any linguistic patterns in input. Therefore, any differences observed between the ‘label’ and ‘no label’ conditions can only be attributed to the *Hebbian learning* that occurred during the training, and which was driven by the presence—or absence—of a wordform in the perisylvian areas, leading to the formation (or resulting in the lack) of an association between such label and the simultaneously presented conceptual patterns.

In the testing phase, each of the trained 30 sensorimotor grounding patterns were presented to the extrasylvian primary areas, *V1 and *M1_L_. Prior to the presentation of each pattern, a global network reset was carried out, upon which the membrane potential of all excitatory and inhibitory cells was set to 0, to ensure that neuronal activity of a previously presented pattern did not affect results. Subsequently, each sensorimotor grounding pattern was presented for two time steps to extrasylvian areas *V1 and *M1_L_ and network responses were recorded during stimulation and the subsequent 28 time steps (30 time steps total). During the two time steps of pattern presentation, no baseline noise was present in any area; during the subsequent 28 time steps of the recording phase, baseline noise stimulation was present in all model areas again, as during training. However, in contrast to the training phase, no uncorrelated white noise was given as input to the perisylvian areas (*A1, *M1_i_) during testing. We computed the estimated mean firing rate of every excitatory neuron in the model in response to each pattern and used this to calculate dissimilarity matrices. More precisely, the estimated instantaneous firing rate *ω_E_*(*e*,*t*) of an excitatory cell *e* at time *t* is defined by:

2.1 $$\tau_{Favg}\cdot \frac{d\omega_{E}(e,t)}{dt}=-\omega_{E}(e,t)+\phi (e,t),$$

where *ϕ*(*e,t*) is the output of cell *e* (either 0 or 1) at time *t* (the definition of *ϕ*(*e,t*) can be found in eqn (3) in [46]). The value *ω_E_*(*e,t*), solution to equation (2.1), is the low-pass-filtered output of cell *e*, integrated using time constant *τ_Favg_* (here, 5). This value provides an estimate (at time *t*) of the cell's recent mean spiking activity. We took the (time discrete) solution to equation (2.1) at time *t* = *t*_30_ (i.e. the value of *ω_E_*(*e,t*_30_), where *t*_30_ = thirty simulation time-steps after pattern-presentation onset) as the estimated mean response (firing rate) of cell *e* to a pattern, and used this value to calculate dissimilarity matrices (see below).

### (g) Data analysis

**Figure 3.**
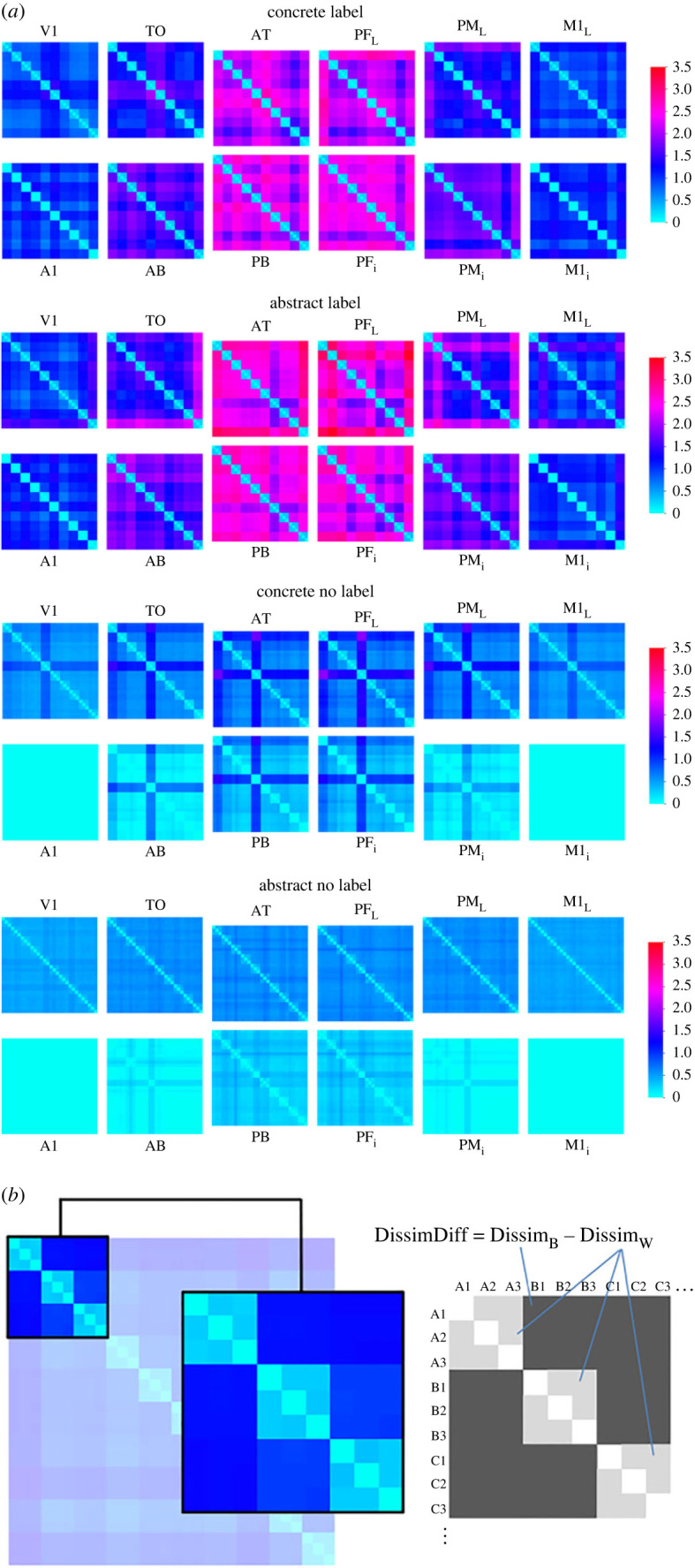
(*a*) Representational dissimilarity matrices (RDMs) for the label condition (top) and no label condition (bottom). In each panel half, concrete concepts are shown on top and abstract concepts on the bottom. In each case, a total of 12 RDMs (one for each model brain area) are shown, arranged in the same way as the 12 brain areas depicted in figure 2*b*. Each RDM is 30 × 30, with three successive positions always reflecting one concept (see also panel *b*). Each cell in a RDM shows, through colour-coding, the dissimilarity between the respective pair of 30 patterns. The dissimilarity measure used was Euclidean distance (with 0 indicating identity). (*b*) Enlargement of a 9 × 9 section of an RDM (i.e. showing 3 out of 10 concepts with letters denoting concepts, and numbers denoting instances within a concept; e.g. A1 denotes instance 1 of concept A etc.) to illustrate the calculation of DissimDiff. We separately calculated overall average dissimilarity for all pairs of conceptual patterns belonging to the same concept (Dissim_W_ – within, light grey) and for all pairs of conceptual patterns belonging to different concepts (Dissim_B_ – between, dark grey). Note that in this enlargement it can be seen that whereas the ‘inner’ (narrow) diagonal has a dissimilarity of 0 (by definition), the ‘within-concept’ dissimilarity values (broader diagonal) in a 3 × 3 grid typically still have a fairly low dissimilarity (lower than outside of this diagonal), but not 0.

**Dissimilarity analysis**. We first recorded the estimated mean firing rate of all model neurons at time *t*_30_ on a per-instance basis (i.e. separately for each of the 30 conceptual grounding patterns). Based on this, we calculated a 30 × 30 dissimilarity matrix showing how dissimilar the network's activity was in response to the different 30 conceptual grounding patterns. We used Euclidean distance as a similarity measure. However, since the 30 patterns consisted of 10 triplets of related patterns (10 concepts consisting of three related patterns each), we further divided the dissimilarity analysis into within-category dissimilarity and between-category dissimilarity (see figure 3*b* for illustration, where letters stand for one semantic concept and 1/2/3 for the three instances). That is, of all the dissimilarity values in the 30 × 30 matrix (only 9 × 9 is shown for illustration), the average of all the light grey values was the within-category dissimilarity (Dissim_W_) and the average of all the dark grey values was the between-category dissimilarity (Dissim_B_). Finally, we used the difference Dissim_B_ -Dissim_W_ (DissimDiff) as a measure of ‘categorical perception’ and hence as a way to quantify how well the model's activity represents the semantic categories inherent in the overlap structure of the conceptual patterns. We, therefore, use the DissimDiff as a way to quantify the semantic category learning performance of the models in our four conditions. Other authors have made a similar proposal, calling this value the global discrimination value [71].

Simulations were carried out on the high-performance computing system of Freie Universität Berlin [72]. Data processing, statistical analyses and figure creation was performed using Python (version 3.7), numpy (version 1.19.2; [73]), pandas (v. 1.1.5; [74]), matplotlib (v. 3.3.2; [75]), seaborn (v. 0.11.0; [76]), scipy (v. 1.5.2; [77]) and statsmodels (v. 0.12.1; [78]). The significance threshold was adjusted to a conservative critical *p* of 0.01.

## 3. Results

In a first step, to illustrate the effect of the presence or absence of linguistic labels during training, we analysed the dissimilarity (Euclidean distance) of model activity in response to input of the 30 grounding patterns to the primary extrasylvian areas. Representational dissimilarity matrices (RDMs) are shown for each of the 12 model areas in figure 3*a*. While within-concept dissimilarity is always quite low (‘narrow diagonals’, i.e. within a light-grey shaded 3 × 3 box as illustrated in figure 3*b*), it can clearly be seen that there are differences depending on the factors semantic-type and label in the between-concept dissimilarity. In general, in the labels condition, between-concept dissimilarity was much higher for both semantic types, indicating that if conceptual patterns were learnt in association with labels the purely perceptuo-motor activity in response to these conceptual patterns on their own was more ‘categorical’, i.e. the differences between the 10 different semantic concepts were amplified. In addition to this strong labelling effect in general, some smaller differences can be seen even in the no label condition between concrete and abstract conceptual patterns: in this case, the between-concept dissimilarity was higher for concrete than for abstract concepts, indicating that with concrete conceptual patterns, ‘categorical’ perceptual processing is more an inherent property of the overlap structure of the grounding patterns, even in the absence of labels. To quantitatively investigate these differences and test for their statistical significance, we conducted further analysis on the DissimDiff values obtained from the dissimilarity matrices in figure 3*a*.

**Figure 4.**
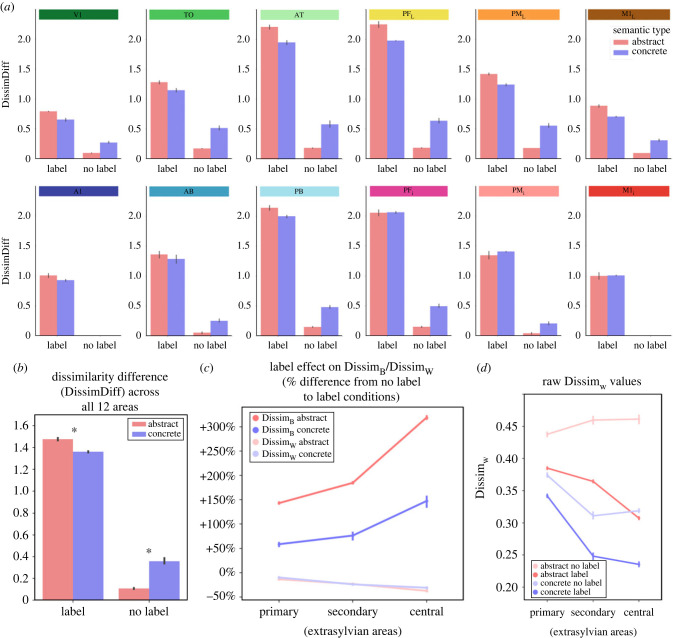
(*a*) DissimDiff (dissimilarity difference) values (i.e. Dissim_B_−Dissim_W_) with factors semantic-type (abstract/concrete) and label (label/no label) across the 12 model areas. This confirmed the visual impression obtained from figure 3. (*b*) For statistical analysis, we collapsed the DissimDiff across all 12 areas and conducted a repeated measures ANOVA with factors semantic-type (2) and label (2). This resulted in a main effect of semantic-type (*F*_1,11_ = 49.9, *p* < 0.0001), a main effect of label (*F*_1,11_ = 14 927, *p* < 0.0001) and a significant interaction between semantic-type and label (*F*_1,11_ = 672.6, *p* < 0.0001). *Post-hoc* Bonferroni-corrected *p*-tests confirmed that without labels, the DissimDiff was larger for concrete than for abstract concepts (*p* < 0.0001), whereas the converse was true with labels (*p* < 0.0001). (*c*) The label effect (percentage change from no label to label conditions) is shown, separately for Dissim_B_ and Dissim_W_. Note that for Dissim_W_, the label effect is significantly larger for abstract than concrete concepts, whereas no such difference is present for Dissim_B_. In addition to this, Dissim_B_ increases towards central (deeper) model areas compared to primary input areas, whereas Dissim_W_ decreases towards central areas, indicating that the representations of perceptuo-motor patterns for the same concept become increasingly more similar to processing ‘depth’ increases (see main text for discussion). Error bars show 95% confidence intervals. (*d*) Because the label effect is much larger for Dissim_B_ than Dissim_W_ (see main text for discussion of possible reasons), we here additionally show raw values for Dissim_W_.

Figure 4*a* shows DissimDiff (dissimilarity difference) values (i.e. Dissim_B_−Dissim_W_) with factors semantic-type (abstract/concrete) and label (label/no label) across the 12 model areas. This confirmed the visual impression obtained from figure 3*a*. For statistical analysis, we collapsed the DissimDiff across all 12 areas (figure 4*b*) and conducted a repeated measures ANOVA with factors semantic-type (2) and labelling (2). This resulted in a main effect of semantic-type (*F*_1,11_ = 49.9, *p* < 0.0001), a main effect of label (*F*_1,11_ = 14 927, *p* < 0.0001) and a significant interaction between semantic-type and label (*F*_1,11_ = 672.6, *p* < 0.0001). *Post-hoc* Bonferroni-corrected *t*-tests confirmed that this interaction was disordinal: without labels, the DissimDiff was larger for concrete than for abstract concepts (*p* < 0.0001), whereas the converse was true with labels (*p* < 0.0001).

We also quantified the label effect in terms of percentage change from no label to label condition (in per cent of the no label value as baseline), separately for Dissim_B_ and Dissim_W_ (figure 4*c*). As we were also interested in how processing changed towards the ‘deeper’ model areas as activity propagated from the primary input areas in extrasylvian cortex towards the central areas, we conducted a repeated measures 2 × 2 × 2 ANOVA on these data with factors between-within (2), semantic-type (2) and centrality (2). The intermediate secondary level is shown in figure 4*c* for illustration; for simplicity of the statistical analyses, we just contrasted the primary areas and the most central ones, i.e. treating centrality as binary factor. We found a significant three-way interaction (*F*_1,11_ = 427, *p* < 0.0001). Running further 2 × 2 ANOVAs separately for each of the two levels of centrality, we found that in both cases there were significant main effects of semantic-type and between-within as well as significant interactions: for primary extrasylvian areas, there was a main effect of between-within (*F*_1,11_ = 8613, *p* < 0.0001), semantic-type (*F*_1,11_ = 1336, *p* < 0.0001) and a significant interaction (*F*_1,11_ = 1570, *p* < 0.0001). For central extrasylvian areas, there was a main effect of between-within (*F*_1,11_ = 5993, *p* < 0.0001), semantic-type (*F*_1,11_ = 783, *p* < 0.0001) and a significant interaction (*F*_1,11_ = 948, *p* < 0.0001). *Post-hoc* Bonferroni corrected *t*-tests showed that in both primary and central areas, the label effect was always larger for abstract than concrete concepts, but with a different polarity for DissimB and DissimW: in primary areas, the label effect on Dissim_B_ was *higher* for abstract (143.0%) than concrete (58.6%) (*p* < 0.0001); in contrast, the label effect on Dissim_W_ was negative, but *more negative* for abstract (−13.5%) than concrete (−9.9%) (*p* < 0.0001). For central areas, the same pattern of results was obtained, but with larger differences: the label effect on Dissim_B_ was again higher for abstract (319.0%) than concrete (147.4%) (*p* < 0.0001), and for Dissim_W_ again more negative for abstract (−37.4%) than concrete (−31.0%) (*p* < 0.0001). Although the effect on DissimW is also significant, it is difficult to see in figure 4*c* owing to the relatively smaller magnitude compared to DissimB. We therefore additionally show raw Dissim_W_ values in figure 4*d*. Bonferroni-corrected *t*-tests on these raw values confirmed that for both concrete and abstract concepts, Dissim_W_ was significantly lower with labels than without (both *p* < 0.0001).

## 4. Discussion

We used a neuroanatomically grounded computational model of language and concept processing in the human brain (figure 2) and trained the model by co-presenting neural patterns (see figure 1 for illustration) thought to correspond to word forms (presented to perisylvian primary areas) and perceptuo-motor grounding patterns indexing real world instances of concepts (to extrasylvian primary areas). Any input supplied to the model during training was only given to the model's primary sensorimotor areas (*A1, *V1, *M1_L_, *M1_i_), whereas our data analysis was based on examining both primary as well as ‘deeper’ areas in the model which correspond to multimodal areas. We asked whether associative learning between categorical instances (grounding patterns) and word forms which relied on biologically realistic Hebbian mechanisms had an effect on the perceptuo-motor processing of these instances. To assess this, the grounding patterns (without their corresponding word form patterns) were presented in a later testing phase and the similarity structure of the elicited activation patterns in the model was assessed. This design allowed us to assess any Whorfian effects of linguistic learning on subsequent perceptuo-motor processes, because, crucially, direct activation of word form patterns was provided only during the training phase, but not during the test phase, on which our data analysis is based.

We found strong evidence that the presence or absence of linguistic labels (word form patterns on extrasylvian areas) during training influenced the processing of conceptual instances (perceptuo-motor processing) during testing and hence evidence for a Whorfian effect of linguistic learning on perceptuo-motor processing, as indicated by two dependent measures. First, we looked at the dissimilarity structure of perceptuo-motor processing (figures 3 and 4), using the dissimilarity difference (DissimDiff) to quantify the degree of simulated categorical 'perception' (in the model). A high DissimDiff indicates that the model's processing of the input of the perceptuo-motor patterns is reflective of the semantic category structure (i.e. patterns which are instances of the same category would be perceived as more similar by the model (hence grouped together), and/or instances of different categories as more dissimilar). Therefore, the DissimDiff is a measure of how strongly the model abstracts away from the specific perceptuo-motor input and instead exhibits processing carved up along the semantic category structure. Our key result is an interaction between semantic type and labelling for this DissimDiff measure (figure 4*b*): when no labels had been supplied during learning, DissimDiff was almost zero for abstract concepts, indicating that hardly any structuring of the input into different concepts occurred. DissimDiff was significantly higher for concrete concepts, indicating that a moderate amount of semantic category learning had occurred, despite the absence of labels during training. By contrast, with labels DissimDiff values became much larger in general and were similar for concrete and abstract concepts (or even slightly higher for abstract concepts). This means that, by adding labels to the concepts, the learning of concrete semantic categories was slightly improved, whereas that of abstract concepts was substantially changed from an almost-absent category structure to a full-fledged one comparable to that of concrete concepts. The Whorfian effect is by far stronger for abstract than for concrete concepts. As hypothesized in the introduction, the fragile categorical conceptual binding for abstract concepts is substantially solidified by the learning and grounding of verbal category terms.

We quantified the label effect separately for Dissim_B_ and Dissim_W_ (figure 4*c*), in percentage change from no label to label conditions (i.e. the no label-label difference in per cent of the no label value as baseline). This shows, firstly, that the presence or absence of labels causes major changes of Dissim_B_, but much smaller (but still significant) ones of Dissim_W_ (see figure 4*d*). Furthermore, the label effect was significantly larger for abstract than concrete concepts. Secondly, in the ‘deeper’ areas of the model (i.e. those further away from the primary sensorimotor areas where stimulation was given), the difference in Dissim_B_ between abstract and concrete concepts became even larger. This indicates that the further away (in the layer hierarchy) from the semantic input (conceptual patterns), the more the network's activity reflects the category structure imposed by the verbal labels, and the more important the role of labels becomes for abstract concepts (compared to concrete ones) in such conceptual structuring. This finding is consistent with neuroimaging results showing that processing abstract words primarily engages inferior frontal and middle temporal regions [79–82]. We see a general tendency for Dissim_W_ to decrease towards central areas, which suggests that the distance between patterns belonging to the same category becomes increasingly smaller as processing depth increases (i.e. these patterns are being treated as increasingly more similar).

In summary, our results clearly demonstrate a positive Whorfian benefit of interlinking conceptual instances with labels. While labels improved category learning performance of the network in general, their addition made a huge difference for abstract category member processing contrasting with a moderate effect on concrete ones. These results lead us to conclude that in order to fully capture the semantic category structure of abstract concepts, the interplay between language and other perceptual and cognitive processes is necessary. Although this conclusion is in line with some of the theoretical proposals mentioned in the introduction, not all of these proposals trace back the result to the mechanism identified here, i.e. the structuring effect of verbal category terms on the learning of abstract concepts with family resemblance relationships.

### (a) Label-induced increase of within-category similarity and between-category dissimilarity

Over and above discussing a general measure of categorical separation induced by language, we here provide evidence for two partly independent processes jointly contributing to such separation. First, as many researchers have emphasized (e.g. [26,30,31]), the learning of words for a set of similar instances increases their representational similarity. Thus objects, actions or scenes from the same category are perceived as more similar to each other. Mechanistically, this effect is owing to a relative increase of model neurons shared by the cell assemblies of the different instances, and, at the same time, relative decrease of neurons specific to individual instance representations. A second process and factor is the label-induced increase of the representational distance between categories. The latter effect appeared to be much more pronounced than the former. Here, we will discuss briefly why this might be so in our present simulations, thereby also discussing aspects of the mechanisms of ‘label addition’ and the change in semantic representation it comes with.

The measure we here use for accessing (dis-)similarity, the Euclidean distance between representational patterns, comes with the property that representations which involve more active neurons come with longer (larger) vectors so that their dissimilarity will be more different from each other than ‘smaller’ representations (with fewer active neurons). In addition, this makes it easy to increase the distance between representations (by adding new active neurons) and thus increase dissimilarity but more difficult to decrease dissimilarity, especially if, as in the present simulations, representational patterns are chosen that strongly overlap in the first place. It may be that when using a different dissimilarity measure (e.g. cosine dissimilarity or a measure normalized for ‘representational size’ or vector length) the results will change in such a way that within- and between-category dynamics appear more comparable in size. We suggest not over-emphasizing this difference between within- and between-category dissimilarities, but instead consider both facts, each of which is based on significant results, as relevant.

As the label-induced ‘moving apart’ of different categories in representational space seems to have received less attention in previous work compared with the ‘moving together’ of representations within the same category, we now turn to the mechanisms underlying the increase of dissimilarity between categories related to language. The addition of different labels to conceptual representations in a network can be modelled in an elementary way by adding a different activation vector – representing the added word form – to each concept vector. For instances (i.e. objects, actions or scenes) from the same semantic category and thus falling under the same categorial term, the same word form vector is added to each instance vector, so that the distance between the resultant vectors remains unchanged. However, if two instances are interlinked with different word form vectors, the difference between the sum vectors (word form + instance vectors) will increase. In this latter case, the sum vectors will be more dissimilar than the instance ones had been on their own. Because all concepts within a category have the same label vector added, the categories move apart from each other in representational space—but not the concepts within each category.

Even though the scenario described above is a simplification (since, for example, nonlinear processes may play an additional role), it might nevertheless capture an important factor moving labelled semantic representations further apart compared with the non-labelled instance and conceptual representations. One may, however, object that the addition of labels in itself does not imply a difference in semantics. Even though representational dissimilarity increases between categories, this would not constitute a semantic effect but purely a consequence of the addition of knowledge about symbol form.

That this latter position is however not fully appropriate can be seen when looking not at dissimilarities, but instead at details of the representational changes in the underlying cell assemblies (an analysis type that we carried out in the context of a previous simulation project [46]). One important aspect here is that the number of neurons responding to all instances of a conceptual category increases when the category label is added. In particular, some neurons previously activated by only 1 or 2 category instances are now active for all the category members. This effect is most prominent for the abstract concepts and their neurons responsive to only 2 of the 3 category members but occurs likewise for single instance neurons of concrete and abstract ones. What this implies is that the addition of a label changes the semantic representation so that previously instance specific perceptual and action related features become attributed to all members of the semantic category. The semantic structure changes as a consequence of the addition of a word form. The mechanism behaves as if some features, which in fact characterize only some category instances, were shared by all category members.

### (b) Limitations and future research needs

In spite of the present attempts to look more closely at the mechanisms by which verbal labels may aid semantic category learning, this work leaves many related questions unanswered. We believe that a likely mechanism is owing to the differing patterns of correlations that (i) the semantic-feature neurons, on the one hand, and (ii) the word form neurons and semantic-feature neurons, on the other, exhibit for concrete and abstract concepts. A proposed explanation is that the relatively stronger correlation between label and semantic neurons (see the Introduction and figure 1 for explanation) for abstract concepts exerts a ‘pull’ on the emerging cell assemblies during training such that the word form neurons play a larger role in the entire semantic cell assembly than they do for concrete concepts. However, to assess this suggestion in detail, one would need to follow the formation of novel cell assemblies for concepts and then semantic conceptual-linguistic representations step by step throughout the learning process. Most importantly, we assume and suggest that the implemented associative learning between concrete and abstract conceptual instantiations and their respective linguistic correlates leads to the formation of neuronal mechanisms that bind the form and meaning of concrete and abstract symbols. These mechanisms are characterized by overlapping neuronal units carrying shared or partially shared semantic features. Actually searching for and eventually documenting these neuronal units after learning in the brain-constrained networks is an important task for future research. A related question is how the learning of conceptual mechanisms affects the neural representations of the individual conceptual instances, the object representations built before label learning started. Would the conceptual representations ‘glue together’ different instance representations or would both coexist in the same model? These are but a few relevant questions to be addressed in future neurocomputational studies.

Although the observed linguistic effects can, following our argument, be explained at the neurobiological level, one may claim that there are cognitive effects of importance without any known neurobiological correlate. For example, infants pay more attention to novel and unfamiliar objects if they are labelled with a category term compared to when they are unlabelled; the mere existence of a verbal label may therefore exert a similar attention-attracting effect as a pointing gesture [39]. In category learning, experiencing several similar objects always paired with the same linguistic label has been shown to cause the learner to pay more attention to the shared features of the objects than the presence of object-specific labels [31,85,86]. However, such attraction of attention and focusing on shared features by verbal category labelling may have a neurobiological mechanism causing it. We here submit that the proposed formation of a strongly connected cell assembly including neurons representing the shared semantic features of concrete concepts is such a mechanism. Such a strongly connected neuron set will, owing to its strong interconnectedness, more easily activate and therefore may provide the mechanism behind the cognitive feature of attention-attraction of labelled object categories.

An obvious simplification introduced in our model is that every concept has only a single label. Realistically, however, most objects can be named at several levels of specificity, for example that of the basic category (e.g. CHAIR, ROBIN) and the larger domain (e.g. FURNITURE, BIRD) [87,88]. A related question concerns the converse situation where a single word form is associated with several conceptual patterns; a recent study [89] found that it is easier for children to learn related meanings of a word form (polysemy) compared to learning unrelated meanings (i.e. homonyms or homophones). Future simulation studies could address semantic hierarchies, polysemy/homonymy and other form-meaning relationships and try to clarify how more complex relationships between form and meaning are neurobiologically realised and affect perceptual and conceptual processing. A related simplification, which we introduced here, was that there was zero overlap between the grounding patterns of different concepts; as we focus on investigating how the differential within-category overlap structure affects the resulting neuronal representations, we avoided additional uncontrolled between-category dis/similarity, as it could perturb or even confound the study results. However, we acknowledge that this is a potential limitation which future simulations could address.

Finally, we would like to emphasize that there are several aspects of human word learning which might play an important role but which are not captured by our current model. These include, for example, the influence of functional object properties on semantic categorization [90], phonological factors such as stress patterns [91] or the classification of words into semantic classes based on distributional and syntactic similarities (syntactic bootstrapping) [92,93] as well as cognitive factors related to the nature of the learning tasks [94] or how easily semantic features of a category can be verbalized [95,96]. One additional aspect not yet addressed here is the cross-linguistic variability of semantics [97]. For example, we do not simulate here cases such as that of colour terms which may partition perceptual space in a language-specific manner—with some languages including one high-frequency symbol for a range of colours (e.g. ‘blue’) and others offering a verbal separation into lighter and darker variants [7]. We note that the present approach to the neurobiology of language and concepts addresses these issues and even provides explanations for the interaction between language and perception processes [10,98].

## 5. Conclusion

A brain constrained neurocomputational simulation study was performed to explore putative brain mechanisms of associating conceptual categories (each constituted by three distinct, but related, grounding patterns) with linguistic labels. We found a clear Whorfian effect of category labels on the processing of conceptual instances: the model's activity in response to perceptuo-motor grounding patterns was modulated depending on whether or not labels had been provided during the earlier training phase. Labels were highly beneficial for semantic category learning performance, and this benefit was more strongly pronounced for abstract compared to concrete concepts and even more so in the deeper-lying semantic ‘hub’ areas of the model than in the primary areas, where stimulation was given. Thus, these effects of linguistic relativity are substantially modulated by the similarity structure of concepts, being more effective and relevant for the formation of abstract concepts with family resemblance structure than for concrete concepts with shared semantic features.

## Data Availability

The datasets supporting this article are available at: https://osf.io/eqhx3/.

## Footnotes

1 We use ‘conceptual’ to refer to purely conceptual processes when no language is involved; by contrast, we use ‘semantic’ when language is involved, which means that a symbol (e.g. a spoken word form, written word, gesture or other symbol) is interlinked with a concept.

2 We sometimes speak about ‘*perceptual*’ features but would like to stress that the same applies to *action* related features too. If the same perceptual (or action) feature is shared by all or most category instances, this feature can be considered *semantic* (e.g., CUCUMBER is [+GREEN]).

3 This section has been adapted from [46,58], as we used the same model architecture and connectivity features here.

4 This section has also been adapted from [46,58], as we used the same model architecture and connectivity features here.

5 This section has been adapted from [46], as we used the same conceptual grounding patterns here.
